# Health-Related Quality of Life and Sleep Quality after 12 Months of Treatment in Nonsevere Obstructive Sleep Apnea: A Randomized Clinical Trial with Continuous Positive Airway Pressure and Mandibular Advancement Splints

**DOI:** 10.1155/2020/2856460

**Published:** 2020-06-30

**Authors:** Lars M. Berg, Torun K. S. Ankjell, Yi-Qian Sun, Tordis A. Trovik, Oddveig G. Rikardsen, Anders Sjögren, Ketil Moen, Sølve Hellem, Vegard Bugten

**Affiliations:** ^1^Department of Clinical Dentistry, Faculty of Health Sciences, UiT The Arctic University of Norway, Tromsø, Norway; ^2^ENT Department, University Hospital in Northern Norway, Tromsø, Norway; ^3^Department of Clinical Medicine, Faculty of Health Sciences, UiT The Arctic University of Norway, Tromsø, Norway; ^4^Center for Oral Health Services and Research, Mid-Norway (TkMidt), Trondheim, Norway; ^5^Department of Clinical and Molecular Medicine, Faculty of Medicine and Health Sciences, NTNU Norwegian University of Science and Technology, Trondheim, Norway; ^6^Department of Community Medicine, Faculty of Health Sciences, UiT The Arctic University of Norway, Tromsø, Norway; ^7^ENT Department, Section for Oral and Maxillofacial Surgery, Arendal Hospital, Arendal, Norway; ^8^Department of Clinical Dentistry, Faculty of Medicine, University of Bergen, Bergen, Norway; ^9^Department of Otorhinolaryngology, Head and Neck Surgery, St. Olav's University Hospital, Trondheim, Norway; ^10^Department of Neuromedicine and Movement Science, Faculty of Medicine and Health Sciences, NTNU Norwegian University of Science and Technology, Trondheim, Norway

## Abstract

In this randomized controlled trial, patients with nonsevere obstructive sleep apnea (OSA) were treated with continuous positive airway pressure (CPAP) or a twin block mandibular advancement splint (MAS). The primary objective was to compare how CPAP and MAS treatments change the health-related quality of life (HRQoL) and self-reported sleep quality of patients after 12 months of treatment. In total, 104 patients were recruited: 55 were allocated to the CPAP treatment group and 49 to the MAS treatment group. We used the SF36 questionnaire to evaluate HRQoL and the Pittsburgh Sleep Quality Index (PSQI) to evaluate sleep quality. All patients were included in the intention-to-treat analyses. These analyses showed improvements in the SF36 physical component score (from 48.8 ± 7.6 at baseline to 50.5 ± 8.0 at follow-up, *p*=0.03) in the CPAP treatment group and in the mental component score (from 44.9 ± 12.1 to 49.3 ± 9.2, *p*=0.009) in the MAS treatment group. The PSQI global score improved in both the CPAP (from 7.7 ± 3.5 to 6.6 ± 2.9, *p*=0.006) and the MAS (8.0 ± 3.1 to 6.1 ± 2.6, *p* < 0.001) treatment groups. No difference was found between the treatment groups in any of the SF36 scores or PSQI global score at the final follow-up (*p* > 0.05) in any analysis. The improvement in the SF36 vitality domain moderately correlated to the improvement in the PSQI global score in both groups (CPAP: |*r*|=0.47, *p* < 0.001; MAS: |*r*|=0.36, *p*=0.01). In the MAS treatment group, we also found a weak correlation between improvements in the SF36 mental component score and PSQI global score (|*r*|=0.28, *p*=0.05). In conclusion, CPAP and MAS treatments lead to similar improvements in the HRQoL and self-reported sleep quality in nonsevere OSA. Improvements in aspects of HRQoL seem to be moderately correlated to the self-reported sleep quality in both CPAP and MAS treatments.

## 1. Introduction

Obstructive sleep apnea (OSA) is a sleep condition associated with reduced health-related quality of life (HRQoL) [1–3]. This may be related to poor sleep quality due to repeated breathing cessations and fragmented sleep [4, 5] or to the characteristics of a typical OSA population, including high body mass index (BMI) and poor subjective health status [2, 6]. Although not universally defined, the term HRQoL is used for describing an individual's somatic, mental, and social well-being, in contrast to the general QoL, which also considers aspects such as economy and living conditions, in addition to health and social status [7]. One widely used questionnaire showing reduced subjective health status and HRQoL in patients with OSA is the “Medical Outcomes Study Short-Form 36-Element Health Survey” (SF36) [1, 8]. Previous studies have shown conflicting effects of OSA treatment on the HRQoL [9–12].

In the adult Norwegian population, the prevalence of OSA is estimated to 16%, of which the majority have nonsevere OSA [13]. In addition to positional therapy in patients with supine-dependent OSA [14, 15], the most common treatments for nonsevere OSA in patients noneligible for surgical intervention are continuous positive airway pressure (CPAP) devices or mandibular advancement splints (MAS) [16, 17]. CPAP treatment effectively reduces the number of pharyngeal soft tissue collapses, which cause the breathing cessations, by creating a pneumatic splint in the upper airways, regardless of OSA severity [18]. By comparison, the efficacy of the MAS treatment is harder to predict without using less accessible procedures such as remotely controlled mandibular protrusion during sleep [19] or drug-induced sleep endoscopy [20, 21], especially in moderate and severe OSA [22, 23]. However, better compliance with MAS treatment makes the overall effectiveness of the two treatments comparable [24] and they are probably equally effective at preventing negative health outcomes associated with OSA [25–27]. Whether the prevention of negative health outcomes with CPAP and MAS treatments is reflected in the HRQoL is uncertain. A meta-analysis by Kuhn et al. [28] presented evidence for the positive effect of CPAP treatment on the HRQoL of patients with OSA when measured using the SF36 questionnaire, while the results regarding MAS treatment were less certain. Thus, more trials investigating the effects of MAS on the HRQoL are needed, preferably in comparison with CPAP treatment [28].

Although commonly used in OSA research, the SF36 questionnaire may not directly evaluate sleep or sleep quality. Questionnaires such as the Pittsburgh Sleep Quality Index (PSQI) evaluate the subjective sleep quality but not the HRQoL [29]. Kang et al. [3] showed that the HRQoL is more associated with sleep quality than with the objectively measured treatment effects on OSA and that sleep quality is also likely to impact the HRQoL. Whether or not the SF36 is sensitive to changes in self-reported sleep quality is not clear, but the SF36 seems to be associated with daytime sleepiness [30], indicating that it may also reflect changes in sleep quality. The aim of this randomized controlled trial was to compare CPAP and MAS after 12 months of treatment in patients with nonsevere OSA, in terms of their HRQoL and self-reported sleep quality, and to investigate the correlation between HRQoL and sleep quality.

## 2. Materials and Methods

### 2.1. Trial Design

This was a two-centered, parallel-arm, randomized, controlled clinical trial, with a 50 : 50 allocation ratio. Blinding of the patients and clinical personnel was not feasible due to the nature of CPAP and MAS treatments.

### 2.2. Participants

Inclusion criteria for participation in the trial were age 20–75 years, apnea-hypopnea-index (AHI) between 10.0 and 29.9, and the ability to protrude the mandible at least 5 mm. Exclusion criteria were severe OSA (AHI ≥30), nasal obstruction, pregnancy, drug abuse, daily use of sedative medication, previous treatment with CPAP or MAS, and preexisting severe psychiatric disorders or somatic health issues interfering with the use of CPAP or MAS, including subjective signs of temporomandibular dysfunction, exaggerated gag reflex, and <10 teeth in the mandible with good periodontal support.

### 2.3. Study Setting and Randomization

Patients participating in the trial were referred from primary healthcare to the ear-nose-throat-departments at the University Hospital of Northern Norway (UNN) in Tromsø, St. Olavs University Hospital (St. Olavs), or Aleris Hospital and Medical Center in Trondheim, Norway. The Aleris Hospital and Medical Center transferred eligible patients to St. Olavs for random allocation and interventions. Two researchers (LMB and TKSA) calibrated all healthcare personnel involved in the trial, according to the study protocol. For random allocation, the patients drew lots from a masked envelope made by one of the researchers (TKSA). Block randomization with 30 lots per block at each study site was used to prevent skewed distribution between treatment groups across seasons and study sites.

### 2.4. Interventions

All patients were screened for OSA overnight at home or at a hotel, using an ambulatory, type 3, polygraphic sleep recording device (Embletta® or Nox T3™, ResMed Norway AS). Sleep technicians manually analyzed the sleep recordings according to the American Academy of Sleep Medicine practice guidelines for diagnostic testing for OSA [31]. Apnea events were defined as ≥90% reduction in respiratory flow lasting ≥10 s. Hypopnea events were defined as ≥50% reduction in respiratory flow lasting ≥10 s, with a simultaneous ≥3% reduction in peripheral blood oxygen saturation from baseline. All patients were medically examined by an otorhinolaryngologist at UNN or St. Olavs Hospitals and were invited to participate in the trial if they met the inclusion criteria. After giving an informed written consent to participate, the patients were randomized to either the CPAP or MAS treatment group. The study protocol for the two treatment groups complied with the recommendations from the Standards of Practice Committee and the Board of Directors of the American Academy of Sleep Medicine [32, 33].

For patients allocated to CPAP treatment, an auto-CPAP device (ResMed®, San Diego, CA, USA) was adapted and calibrated by a sleep technician. A nose mask or face mask was used, based on the needs and preferences of the individual patient. Patients returned for a follow-up visit 4 months after treatment onset. A sleep technician downloaded efficacy data from the CPAP device, made necessary adjustments to the CPAP device, and gave a motivational talk to advocate further use of the CPAP device.

For patients allocated to MAS treatment, an intraoral examination followed by a bite registration using the George Gauge™ (Scheu-Dental GmbH, Iserlohn, Germany) and an impression of the dentition was made by a dentist. The impressions and bite registration were sent to a dental technician for the production of the MAS (Respire Medical, New York, NY, USA, or SomnoDent®, Sydney, NSW, Australia). All MAS had the same twin block design, although produced by two different manufacturers. The MAS was set to 60–65% of maximum mandibular protrusion at treatment onset and titrated to maximal comfortable mandibular protrusion after two to three weeks by the dentist. Patients in the MAS treatment group also returned for a follow-up visit 4 months after treatment onset. A sleep technician performed and analyzed a new overnight polygraphic sleep recording and gave a motivational talk to advocate further use of the MAS. The MAS was used during the overnight polygraphic sleep recording at follow-up. Necessary adjustments to the MAS were subsequently made by a dentist.

About 12 months after the treatment was initiated, all patients returned for a final follow-up visit, during which efficacy data were downloaded from the CPAP device for the CPAP treatment group and a new overnight polygraphic sleep recording was performed for the MAS treatment group while using the MAS.

### 2.5. Outcomes

The HRQoL was evaluated at baseline and at both follow-up visits using the SF36 questionnaire (version 2). This questionnaire is a widely used, multipurpose, generic, and validated questionnaire, consisting of 36 questions that measure the relative burden of disease and health conditions [28, 34]. The SF36 yields eight HRQoL domains scored on a 0–100 scale, where zero represents the worst and 100 represents the best HRQoL. The 0–100 scales are standardized into norm-based scores to allow direct comparison among different domains and in relation to the general population. A norm-based score more or less than 50 represents a better or worse HRQoL, respectively, than the average general population. The eight norm-based domains were united into one physical and one mental aggregated health scale [34]. We present the norm-based scores, while the 0–100 scales are presented in Supplementary Tables S1–S3.

The self-reported sleep quality was measured using the PSQI questionnaire at baseline and at both follow-ups. The PSQI is a validated questionnaire consisting of 19 questions, assessing seven aspects of sleep quality: subjective sleep quality, sleep latency, sleep duration, habitual sleep efficiency, sleep disturbances, use of sleeping medicine, and daytime dysfunction. These aspects were transformed into a sum score ranging from zero to 21 points, where good sleep quality is defined as ≤5 points [29].

To make results in the SF36 and PSQI comparable, the reliable change index (RCI) was used to calculate a standardized change in the SF36 domains and PSQI global score from baseline to final follow-up in each individual patient, as described by Jacobson and Truax [35]. The RCI was calculated using the test-retest figures by Stavem et al. [36] and Buysse et al. [29] and the pretreatment standard deviation in each SF36 domain and PSQI global score. A |RCI-value| >1.96 indicated a statistically significant change from baseline on a 5% significance level, i.e., a change not likely to occur due to test-retest variations. Statistical significant changes in the RCI corresponded to clinically significant changes, with a bigger |RCI value| also indicating a bigger clinical change [35]. The RCI enabled correlation analysis between changes in the SF36 domain scores and those in the PSQI global score after OSA treatment.

Demographic characteristics of the patients were collected through a questionnaire at the time of the medical examination prior to treatment. Compliance with treatment was self-reported at the final follow-up and defined as using the CPAP device or MAS for more than 4 hours per night, more than 70% of the nights [37, 38].

### 2.6. Sample Size

Based on an expected 10% difference between the treatment groups in the SF36 domain scores at final follow-up [39] and a common standard deviation within groups at 20%, a sample size of 69 patients in each treatment group at the final follow-up was needed to detect between-group differences at a 5% significance level and reaching 80% power in a two-tailed *t*-test. Similarly, to detect a difference in the PSQI global score between the treatment groups, 45 patients were needed in each treatment group, based on an expected difference of 15% between groups, and a 25% standard deviation within groups at the final follow-up.

### 2.7. Statistical Analysis

Both intention-to-treat (all included patients) and per-protocol (patients compliant to treatment at final follow-up) analyses are presented. Differences between the two treatment groups regarding average SF36 scores and the PSQI global score at final follow-up were analyzed using multivariable linear regression and adjusted for age, BMI, sex, smoking, AHI, and the SF36 domain/PSQI global score at baseline. A paired sample *t*-test was used to analyze changes from baseline at the final follow-up within each treatment group. Mann–Whitney *U-*test and paired sample Wilcoxon test were used to compare changes in the AHI between and within treatment groups. The correlation between significantly changed SF36 domain scores and the PSQI global score was examined using Pearson correlation analysis on RCI values. Larger RCI values represented larger changes from baseline at the final follow-up in the respective scales. Differences in the number of patients with improved scores between treatment groups were analyzed using Pearson chi-square and Fisher's exact tests. Any missing entries in the SF36 questionnaire were replaced in accordance with Ware et al. [34].

Missing entries in the PSQI questionnaire were replaced through multiple imputations, as recommended by CONSORT 2010 [40, 41]. Data missing at the final follow-up in patients who discontinued treatment were replaced by data from baseline or the 4-month follow-up for the intention-to-treat analysis.

All statistical analyses were performed using SPSS 26 statistical software package (IBM Corp, Armonk, NY, USA) and a two-sided *p* < 0.05 was considered statistically significant.

### 2.8. Ethical Approval

This randomized controlled trial was approved by the Norwegian Regional Committee for Medical and Health Research Ethics, REC Central (registration #2014/956), and is registered in ClinicalTrials.gov (registration #NCT02953028).

## 3. Results

### 3.1. Recruitment and Participant Flow

**Figure 1:**
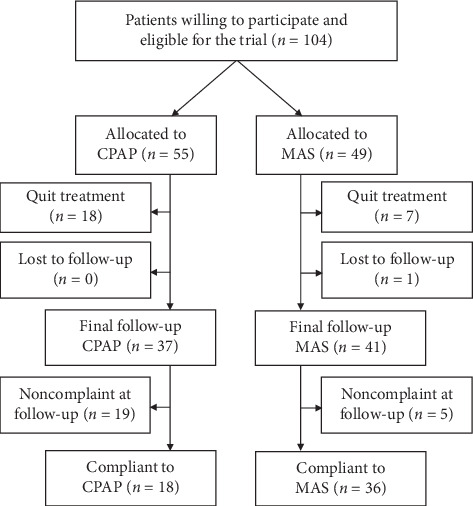
Patient flowchart.

The patients were recruited between October 2014 and February 2018. Informed written consent to participate was obtained from 104 patients, of which 55 were allocated to the CPAP treatment group and 49 to the MAS treatment group. The final follow-up visit occurred between 10 and 20 months (median 13 months) after treatment onset and completed by October 2019.  Figure 1 illustrates the distribution and flow of patients in the study.

At the final follow-up, 18 patients (32.7%) in the CPAP treatment group and seven (14.3%) in the MAS treatment group had quit treatment, all reporting not being compliant to treatment. One patient (2.0%) in the MAS treatment group withdrew from the trial before the final follow-up for no specific reason, being compliant to treatment up to that point. There were more smokers (35.1%) among patients discontinuing treatment before the final follow-up than among those continuing treatment (11.9%) (*p*=0.005). Patients discontinuing treatment did not differ from the remaining study population in any other way at baseline. Among patients discontinuing treatment, no improvement was found in any SF36 domain at a group level, but the PSQI global score was improved from 7.8 to 6.6 points (*p*=0.03).

### 3.2. Number of Participants Analyzed

All recruited patients were included in the intention-to-treat analysis of SF36 and PSQI scores. In the per-protocol analysis, 18 patients (32.7%) in the CPAP treatment group and 36 (73.5%) in the MAS treatment group were regarded as compliant and included in the analysis. When only including cases with complete data, i.e., excluding patients with imputations in the scores of any questionnaire at the final follow-up, 34 and 39 patients were included in the CPAP and MAS treatment groups, respectively (Supplementary Table S4). The baseline characteristics of all recruited patients are presented in Table 1 and were similar between treatment groups.

### 3.3. SF36 Domains

At the final follow-up, there were no statistically significant differences between the CPAP and MAS treatment groups in any of the SF36 domains or component scores. This was the case for both the intention-to-treat (Table 2) and per-protocol analyses (Table 3). In the intention-to-treat analysis, the SF36 role-physical (*p*=0.04) and vitality (*p*=0.006) domains and the physical component score (*p*=0.03) were improved in the CPAP treatment group, while the SF36 vitality (*p* < 0.001) and social functioning (*p*=0.04) domains and the mental component score (*p*=0.009) were improved in the MAS treatment group. In the per-protocol analysis, the SF36 vitality (*p*=0.03) and social functioning domains (*p*=0.02) and the mental component score (*p*=0.003) were improved in the CPAP treatment group, while the SF36 vitality (*p* < 0.001), social functioning (*p*=0.02), and mental health (*p*=0.02) domains and the mental component score (*p*=0.003) were improved in the MAS treatment group.

### 3.4. PSQI Global Score and AHI

At the final follow-up, there were no statistically significant differences between the CPAP and MAS treatment groups in the PSQI global score, which was improved by both CPAP (*p*=0.006) and MAS (*p* < 0.001) treatments based on both the intention-to-treat (Table 2) and per-protocol analysis (CPAP: *p*=0.03; MAS: *p* < 0.001; Table 3).

In the per-protocol analysis, the median AHI at the final follow-up was significantly better (*p* < 0.001) in the CPAP (0.9, 0.7–1.4) than in the MAS treatment group (10.1, 6.1–16.5). Both treatment groups showed significant improvements in the AHI from baseline at the final follow-up (*p* < 0.001).

### 3.5. Correlations between SF36 Domain Scores and PSQI Global Score

The improvement in the SF36 vitality domain score was moderately correlated to that in the PSQI global score in both the CPAP (|*r*|=0.47, *p* < 0.001) and MAS treatment groups (|*r*|=0.36, *p*=0.01). In the latter, there was a weak correlation between improvements in the SF36 mental component score and PSQI global score (|*r*|=0.28, *p*=0.05). In the per-protocol analysis, the improvement in the SF36 vitality domain was strongly correlated with that in the PSQI global score in the CPAP treatment group (|*r*|=0.51, *p*=0.03). Other SF36 domains or component scores with significant improvement after treatment were not correlated to the improvement in the PSQI global score. The number of patients in each treatment group showing improvement according to the RCI in SF36 domains and the PSQI global score is presented in Table 4 (intention-to-treat) and Table 5 (per-protocol).

## 4. Discussion

In this randomized controlled trial, we compared changes in the HRQoL and self-reported sleep quality after 12 months of CPAP or MAS treatment in patients with nonsevere OSA. All patients in this trial were recruited after a referral from primary healthcare services. The randomization procedure was successful and created comparable groups at baseline. Baseline variables were similar in the intention-to-treat and per-protocol analyses. It is likely that the included patients are representative of patients with nonsevere OSA, who do not need nasal or pharyngeal surgical corrections, and are referred to Norwegian public and private hospitals. The patients had higher BMI and worse self-reported general health than the Norwegian general population at baseline [42, 43], but were comparable to OSA populations in other recent Norwegian studies [2, 44]. The mean values for all SF36 domains at baseline were lower than but within one standard deviation from the mean values of the Norwegian general population [45]. The PSQI global score was above the cutoff for good sleep quality in both treatment groups, which is defined as a global score of 5 points or below according to the developers of the PSQI [29].

### 4.1. SF36 and PSQI

Both CPAP and MAS treatments improved the SF36 vitality domain, which is in line with the findings of the meta-analysis by Kuhn et al. [28]. In the intention-to-treat analysis, CPAP treatment also improved the SF36 role-physical domain and physical component scores, while MAS treatment improved the SF36 social functioning domain and mental health component scores. In the per-protocol analysis, both CPAP and MAS treatments improved the SF36 domains of vitality and social functioning and the mental component score, while only MAS treatment improved the SF36 mental health domain score. The PSQI global score was also significantly improved after 12 months of treatment in both treatment groups and in both the intention-to-treat and per-protocol analyses.

Although improvements in several SF36 domain scores were found, the SF36 is a generic questionnaire and thus not specific to OSA [1]. Hence, SF36 does not directly measure sleep quality [34] and may not be representative of patients with sleep disorders, such as OSA. Previous studies have shown lacking association between OSA severity (measured in AHI) and the impairment in the HRQoL [3, 6]. Besides, it is not clear whether it is unspecific symptoms overlapping with OSA symptoms or true OSA symptoms that lower the SF36 scores among OSA patients [2]. Communicating the OSA diagnosis to patients may itself improve the HRQoL, with no other treatment [46]. This suggests that the change in SF36 domain scores after treatment of OSA may be attributed to the patients being diagnosed and cared for or to placebo effects associated with OSA treatment, and not solely to the effect of CPAP or MAS treatment.

Nevertheless, patients compliant to CPAP and MAS treatments showed greater improvements in their HRQoL than those noncompliant to treatment. Furthermore, the SF36 vitality domain, which showed the biggest improvement in both treatments, likely reflects the patient's sleep quality, considering that the questions composing this domain are closely associated with daytime sleepiness: (1) “Did you feel full of life?” (2) “Did you have a lot of energy?” (3) “Did you feel worn out?” and (4) “Did you feel tired?”. The correlation between the SF36 vitality domain score and PSQI global score also indicates that this SF36 domain is the one most likely to respond to changes in sleep quality, but this association does not imply a causal link between HRQoL and sleep quality.

Since the improvement in the physical component score in the CPAP treatment group was found in the intention-to-treat but not in the per-protocol analysis, this score is likely to have improved in some patients noncompliant to treatment. If so, the physical component score improved in noncompliant patients for reasons other than those in patients effectively treated with CPAP or MAS. For example, it is possible that some noncompliant patients started doing physical exercises or reduced their body weight after getting the OSA diagnosis, to compensate for not using CPAP or MAS [47, 48]. If doing so, it is likely that they improved the subjective sleep quality as well [49].

### 4.2. Compliance and AHI

Difficulties in maintaining compliance with treatment is a known challenge in the treatment of nonsevere OSA, especially in CPAP treatment [38, 50]; so, lower compliance in the CPAP than in the MAS treatment group could be expected [23, 26]. However, the compliance with CPAP in this study was lower than expected [51–54]. Discomfort related to the CPAP mask, choking sensation, and xerostomia were the most reported reasons for noncompliance. Nevertheless, investigating the reasons for the particularly poor compliance with CPAP treatment in this study was beyond the scope of this article. Based on the findings in the present trial, differences in compliance between treatment groups should be considered when planning and evaluating the success of CPAP and MAS treatments [26, 55].

Despite an unambiguous better AHI improvement in the CPAP treatment group, it is worth noticing that MAS treatment showed benefits similar to CPAP treatment regarding the HRQoL and self-reported sleep quality, even when comparing compliant patients only. Furthermore, RCI analyses showed that the number of patients experiencing an improvement in individual SF36 domain scores and PSQI global score was similar between the treatment groups. Changes according to the RCI are not likely to occur due to test-retest variations in repeated completion of the respective questionnaires. Therefore, a statistically significant change in the RCI also represents a clinically significant change on a patient level [35]. This suggests that MAS treatment provide subjective benefits equal to that from CPAP in the treatment of nonsevere OSA and may be considered first-line treatment in patients who are more motivated for MAS treatment than CPAP treatment.

### 4.3. Risk and Handling of Bias

In the current study, the results in the per-protocol analysis only included patients who used the CPAP device or MAS for more than 4 hours, 70% of the nights. Therefore, results from the per-protocol analysis should be representative of the efficacy of CPAP and MAS treatments in improving the HRQoL and sleep quality. However, in addition to having a small number of included patients, the per-protocol analysis is prone to bias due to exclusion of patients discontinuing or being semicompliant to treatment. Dropout analyses showed that patients discontinuing treatment had rather similar baseline characteristics to patients compliant to treatment. Hence, the treatment groups should be comparable in the per-protocol analysis. Although similarities between discontinuing and compliant patients indicate a low risk of dropout bias, it is possible that some patient characteristics other than those reported in this study were different between these patients.

In contrast to the per-protocol analysis, the intention-to-treat analysis maintains the strengths of the randomization, thus being less prone to bias. The disadvantage of the intention-to-treat analysis is the inclusion of noncompliant patients, some of whom were not using their assigned treatment device at all. Therefore, this analysis might underestimate the true effects of CPAP and MAS treatments on the HRQoL and sleep quality, especially in the CPAP treatment group where only 32.7% of the patients were regarded compliant to treatment. The discontinuing patients in this study had no significant change in any of the SF36 domains, but the noncompliant patients had a barely significant improvement in the PSQI global score. Although unlikely, it is unclear whether this improvement is clinically significant on a group level.

The CPAP and MAS treatment groups were fairly similar at the final follow-up when comparing their HRQoL and sleep quality, but the two groups seemed to be more similar in the per-protocol than the intention-to-treat analysis. This could be because much fewer patients were included in the per-protocol analysis than the calculated number needed to show differences between treatment groups. To reduce the influence of potential confounders and bias, linear regression models were used to adjust the differences between the two treatment groups for baseline variables. Only minor changes were found regarding the differences between CPAP and MAS treatments in both SF36 scores and PSQI global score. Adjusting for baseline variables did not alter the lack of significant differences between the treatment groups.

Since this was an unblinded trial, risk of bias from the clinical handling of the patients was unavoidable. To avoid biasing common variables between the treatment groups, all clinical personnel were instructed to approach the patients in a standardized fashion. However, some of the possible biases from the lack of blinding are inherently entangled with the provided treatment. Thus, differences in the handling of patients related to the characteristics of CPAP and MAS treatments are inevitable in both clinical research and clinical practice.

## 5. Limitations

A major limitation of this study is the risk of being underpowered. Power analysis prior to the patient recruitment suggested that 69 patients in each treatment group were needed to show differences between CPAP and MAS treatments in the SF36 physical and mental component scores. The number of patients in the trial was the maximum that could be recruited within the time span of this trial, but may not have been sufficient to show differences between the two treatments, especially in the per-protocol analysis. Since 66% of the patients in the CPAP treatment group were considered noncompliant, approximately three times more patients should have been recruited to the study to find any differences between treatment groups in the per-protocol analysis according to the power calculation for the SF36 domain scores. The low number of patients in the per-protocol analysis suggested that a nonparametric test was more suitable to test the statistical differences between treatment groups. However, no differences were found when using the Mann–Whitney *U*-test instead of the regression analysis.

It is plausible that even with 69 patients in each treatment group, the null hypothesis would still not be falsified in this trial due to the similar results found between treatment groups. Moreover, the number of patients in the trial was larger than the calculated number needed to show differences in the PSQI global score between treatment groups; however, no significant differences were found between treatment groups in this score either.

Using self-reported compliance data is another limitation of the study. Objective compliance data were not available for the MAS treatment group, and thus, self-reported data were used for both treatment groups to enable the comparison of compliance data. In contrast to the compliance data downloaded from the CPAP device, four patients misreported themselves as compliant, all of whom had objective CPAP usage very close to 4 hours in 70% of the nights and slightly overestimated their compliance. Previous studies have shown that patients using MAS only slightly overestimate their compliance compared to the objectively measured compliance after 12 months of treatment [56]. Thus, the self-reported compliance is likely comparable between the CPAP and MAS treatment groups at the final follow-up.

## 6. Conclusions

In summary, both CPAP and MAS treatments seemed to improve vitality and mental aspects of the HRQoL, as well as the self-reported sleep quality in patients with nonsevere OSA. In this study, HRQoL and self-reported sleep quality were similar after 12 months of CPAP and MAS treatments. Between improvements in aspects of HRQoL and self-reported sleep quality, a moderate to strong correlation was found after CPAP treatment, while a weak to moderate correlation was found after MAS treatment.

## Figures and Tables

**Table 1:** Patient characteristics at baseline, *n* (%).

Baseline variables	Total (*n* = 104)	CPAP (*n* = 55)	MAS (*n* = 49)
Age at inclusion^a^	51.7 (9.8)	53.3 (10.2)	49.6 (9.0)

BMI at inclusion^a^	31.5 (6.7)	30.8 (6.2)	32.4 (7.2)

AHI at inclusion^b^	17.6 (13.2–23.5)	18.1 (15.3–24.6)	16.3 (12.4–23.0)

Sex			
Female	37 (35.6)	17 (30.9)	20 (40.8)
Male	67 (64.4)	38 (69.1)	29 (59.2)

Marital status			
Cohabitating	81 (77.9)	44 (80.0)	37 (75.5)
Living alone	23 (22.1)	11 (20.0)	12 (24.5)

Allergic rhinitis			
Yes	17 (16.3)	9 (16.4)	8 (16.3)
No	87 (83.7)	46 (83.6)	51 (83.7)

Self-reported health			
Good-excellent	29 (27.9)	16 (29.1)	13 (26.5)
Poor-fair	75 (72.1)	39 (70.9)	36 (73.5)

Education level			
College or university	50 (48.1)	23 (41.8)	27 (55.1)
Other education	54 (51.9)	32 (58.2)	22 (44.9)

Alcohol consumption			
≤1 time/week	83 (79.8)	43 (78.2)	40 (81.6)
>1 time/week	21 (20.2)	12 (21.8)	9 (18.4)

Smoking status			
Nonsmoking	83 (79.8)	41 (74.5)	42 (85.7)
Smoking	21 (20.2)	14 (25.5)	7 (14.3)

CPAP: continuous positive airway pressure; MAS: mandibular advancement splint; BMI: body mass index (kg/m^2^); AHI: apnea-hypopnea index. ^a^Mean (standard deviation); ^b^median (25–75 percentiles). Allergic rhinitis: any respiratory complaints attributed to allergic rhinitis; smoking: current occasional or daily use of smoking tobacco.

**Table 2:** SF36 domains (norm-based scales) and PSQI global score at baseline and final follow-up (12 months), based on intention-to-treat analysis.

SF36 domains	Baseline		Follow-up		Adj. difference (95% CI)^§^	*p* ^§^
	CPAP (*n* = 55)	MAS (*n* = 49)	CPAP (*n* = 55)	MAS (*n* = 49)		
Physical functioning	48.2 (8.9)	47.5 (8.2)	50.0 (8.4)	48.2 (9.6)	−1.6 (−4.4–1.1)	0.23
Role-physical	49.6 (6.8)	48.6 (8.3)	51.4^*∗*^ (6.3)	49.7 (8.2)	−1.9 (−4.4–0.5)	0.13
Bodily pain	49.2 (11.4)	46.3 (10.1)	50.0 (10.7)	46.8 (11.4)	−1.0 (−4.5–2.5)	0.59
General health	45.4 (9.8)	45.8 (10.6)	48.2 (9.9)	46.9 (10.7)	−1.1 (−4.1–2.0)	0.48
Vitality	42.8 (11.4)	39.8 (10.1)	47.4^*∗*^ (10.8)	47.7^*∗*^ (10.5)	2.0 (−1.9–5.9)	0.32
Social functioning	44.5 (12.4)	42.2 (12.2)	47.5 (12.4)	46.5^*∗*^ (10.2)	0.3 (−4.1–4.6)	0.91
Role-emotional	48.4 (8.1)	48.4 (8.1)	49.5 (8.6)	49.6 (7.4)	0.2 (−2.7–3.0)	0.91
Mental health	47.7 (10.0)	47.8 (11.4)	48.9 (11.7)	50.4 (8.5)	1.9 (−1.8–5.7)	0.30
Physical component score	48.8 (7.6)	47.0 (9.4)	50.5^*∗*^ (8.0)	47.5 (10.4)	−1.8 (−4.1–0.5)	0.13
Mental component score	45.6 (10.3)	44.9 (12.1)	47.8 (12.3)	49.3^*∗*^ (9.2)	2.5 (−1.3–6.3)	0.20
PSQI global score	7.7 (3.5)	8.0 (3.1)	6.6^*∗*^ (2.9)	6.0^*∗*^ (2.6)	−0.8 (−1.8–0.1)	0.09

CPAP: continuous positive airway pressure; MAS: mandibular advancement splint; PSQI: Pittsburgh Sleep Quality Index; SF36: Medical Outcomes Study Short-Form 36-Element Health Survey; SF36 domains and PSQI global score: mean (standard deviation). ^*∗*^Statistically significant change from baseline to follow-up within treatment group, paired *t*-test (*p* < 0.05). ^§^Difference between MAS and CPAP treatment groups at follow-up, based on linear regression analysis adjusted for baseline variables (age, BMI, sex, smoking, baseline AHI, and the baseline SF36 domain/PSQI global score), reference group: CPAP.

**Table 3:** SF36 domains (norm-based scales) and PSQI global score at baseline and final follow-up (12 months), based on per-protocol analysis (compliant patients only).

SF36 domains	Baseline		Follow-up		Adj. difference (95% CI)^§^	*p* ^§^
	CPAP (*n* = 18)	MAS (*n* = 36)	CPAP (*n* = 18)	MAS (*n* = 36)		
Physical functioning	47.9 (9.0)	48.3 (7.7)	49.8 (8.3)	49.7 (9.1)	−0.8 (−4.5–2.8)	0.64
Role-physical	49.4 (6.3)	48.4 (9.0)	50.4 (7.2)	50.2 (8.3)	−0.3 (−4.1–3.6)	0.89
Bodily pain	48.4 (12.4)	46.7 (10.3)	47.7 (11.7)	46.9 (11.5)	0.1 (−5.8–6.0)	0.97
General health	45.9 (10.2)	46.9 (10.5)	49.1 (10.5)	49.2 (10.1)	−0.4 (−4.9–4.2)	0.88
Vitality	43.2 (13.7)	39.8 (10.1)	51.0^*∗*^ (9.4)	50.2^*∗*^ (8.4)	0.0 (−5.3–5.2)	0.99
Social functioning	46.6 (12.7)	41.9 (13.0)	51.8^*∗*^ (8.7)	47.7^*∗*^ (9.3)	−1.7 (−6.9–3.5)	0.51
Role-emotional	49.5 (7.3)	48.0 (8.8)	51.3 (6.4)	50.0 (7.4)	0.3 (−3.6–4.1)	0.89
Mental health	49.2 (8.8)	47.2 (12.4)	53.3 (8.1)	51.5^*∗*^ (8.2)	−0.8 (−5.1–3.5)	0.71
Physical component score	47.7 (8.3)	47.9 (9.1)	48.2 (8.7)	48.5 (10.0)	0.0 (−3.6–3.7)	0.99
Mental component score	47.6 (9.6)	44.1 (12.5)	53.2^*∗*^ (4.9)	50.5^*∗*^ (8.0)	−0.2 (−3.9–3.4)	0.91
PSQI global score	7.1 (3.4)	7.7 (3.3)	5.7^*∗*^ (2.3)	5.4^*∗*^ (2.5)	−0.6 (−1.7–0.5)	0.25

CPAP: continuous positive airway pressure; MAS: mandibular advancement splint; PSQI: Pittsburgh Sleep Quality Index SF36: Medical Outcomes Study Short-Form 36-Element Health Survey; SF36 domains and PSQI global score: mean (standard deviation). ^*∗*^Statistically significant change from baseline to follow-up within treatment group, paired *t*-test (*p* < 0.05). ^§^Difference between MAS and CPAP treatment groups at follow-up, based on linear regression analysis adjusted for baseline variables (age, BMI, sex, smoking, baseline AHI, and the baseline SF36 domain/PSQI global score), reference group: CPAP.

**Table 4:** Number of patients with improved SF36 scores according to RCI (>1.96) and PSQI global score according to RCI (<−1.96), based on intention-to-treat analysis.

	Significantly improved HRQoL or sleep quality		
	CPAP (*n* = 55)	MAS (*n* = 49)	*p*
Physical functioning	16.4% (9/55)	12.2% (6/49)	0.55
Role-physical	12.7% (7/55)	12.2% (6/49)	0.94
Bodily pain	7.3% (4/55)	10.2% (5/49)	0.73^F^
General health	21.8% (12/55)	28.6% (14/49)	0.43
Vitality	36.4%^*∗*^ (20/55)	44.9%^*∗*^ (22/49)	0.38
Social functioning	12.7% (7/55)	18.4% (9/49)	0.43
Role-emotional	9.1% (5/55)	12.2% (6/49)	0.60
Mental health	14.5% (8/55)	22.4% (11/49)	0.30
Physical component	10.9% (6/55)	8.2% (4/49)	0.75^F^
Mental component	18.2% (10/55)	20.4%^*∗*^ (10/49)	0.77
PSQI global score	18.2% (10/55)	32.7% (16/49)	0.09

HRQoL: health-related quality of life; RCI: reliable change index; PSQI: Pittsburgh Sleep Quality Index (global score); SF36: Short Form 36 (8 domains + 2 aggregated scales); CPAP: continuous positive airway pressure; MAS: mandibular advancement splint; *P*: Pearson chi-square test; F: Fisher's exact test. ^*∗*^Significant correlation to PSQI global score.

**Table 5:** Number of patients with improved SF36 scores according to RCI (>1.96) and PSQI global score according to RCI (<−1.96), compliant patients only.

	Significantly improved HRQoL or sleep quality		
	CPAP (*n* = 18)	MAS (*n* = 36)	*p*
Physical functioning	11.1% (2/18)	11.1% (4/36)	1.00^F^
Role-physical	5.6% (1/18)	13.9% (5/36)	0.65^F^
Bodily pain	5.6% (1/18)	11.1% (4/36)	0.66^F^
General health	27.8% (5/18)	36.1% (13/36)	0.54
Vitality	38.9%^*∗*^ (7/18)	50.0% (18/36)	0.44
Social functioning	5.6% (1/18)	19.4% (7/36)	0.26^F^
Role-emotional	11.1% (2/18)	13.9% (5/36)	1.00^F^
Mental health	16.7% (3/18)	25.0% (9/36)	0.73^F^
Physical component	5.6% (1/18)	11.1% (4/36)	0.66
Mental component	22.2% (4/18)	22.2% (8/36)	1.00^F^
PSQI global score	16.7% (3/18)	33.3% (12/36)	0.20

HRQoL: health-related quality of life; RCI: reliable change index; PSQI: Pittsburgh Sleep Quality Index (global score); SF36: Short Form 36 (8 domains + 2 aggregated scales); CPAP: continuous positive airway pressure; MAS: mandibular advancement splint; *P*: Pearson chi-square test; F: Fisher's exact test. ^*∗*^Significant correlation to PSQI global score.

## Data Availability

The datasets generated and/or analyzed during the current study are not publicly available due to planned publications based on data included in the present datasets, but are available from the corresponding author on reasonable request.

## Supplementary Materials

Supplementary Materials Supplementary tables presenting SF36 domain scores at baseline and follow-up according to the 0−100 scales. Intention-to-treat analyses (Table S1), per-protocol analyses (Table S2), and complete case analyses (Table S3) are provided. Table S4: present complete case analyses of the norm-based SF36 domain scores and PSQI global score at baseline and follow-up.
