# Corrigendum to “Health-Related Quality of Life and Sleep Quality after 12 Months of Treatment in Nonsevere Obstructive Sleep Apnea: A Randomized Clinical Trial with Continuous Positive Airway Pressure and Mandibular Advancement Splints”

**DOI:** 10.1155/2021/9767184

**Published:** 2021-05-26

**Authors:** Lars M. Berg, Torun K. S. Ankjell, Yi-Qian Sun, Tordis A. Trovik, Oddveig G. Rikardsen, Anders Sjögren, Ketil Moen, Sølve Hellem, Vegard Bugten

**Affiliations:** ^1^Department of Clinical Dentistry, Faculty of Health Sciences, UiT the Arctic University of Norway, Tromsø, Norway; ^2^ENT Department, University Hospital in Northern Norway, Tromsø, Norway; ^3^Department of Clinical Medicine, Faculty of Health Sciences, UiT the Arctic University of Norway, Tromsø, Norway; ^4^Center for Oral Health Services and Research, Mid-Norway (TkMidt), Trondheim, Norway; ^5^Department of Clinical and Molecular Medicine, Faculty of Medicine and Health Sciences, NTNU Norwegian University of Science and Technology, Trondheim, Norway; ^6^Department of Community Medicine, Faculty of Health Sciences, UiT the Arctic University of Norway, Tromsø, Norway; ^7^ENT Department, Section for Oral and Maxillofacial Surgery, Arendal Hospital, Arendal, Norway; ^8^Department of Clinical Dentistry, Faculty of Medicine, University of Bergen, Bergen, Norway; ^9^Department of Neuromedicine and Movement Science, Faculty of Medicine and Health Sciences, NTNU Norwegian University of Science and Technology, Trondheim, Norway; ^10^Department of Otorhinolaryngology, Head and Neck Surgery, St. Olav's University Hospital, Trondheim, Norway

In the article titled “Health-Related Quality of Life and Sleep Quality after 12 Months of Treatment in Nonsevere Obstructive Sleep Apnea: A Randomized Clinical Trial with Continuous Positive Airway Pressure and Mandibular Advancement Splints” [1], the authors identified an error in Section 2.4 as follows:

“Hypopnea events were defined as ≥50% reduction in respiratory flow lasting ≥10 s, with a simultaneous ≥3% reduction in peripheral blood oxygen saturation from baseline” should be corrected to “Hypopnea events were defined as ≥30% reduction in respiratory flow lasting ≥10 s, with a simultaneous ≥3% reduction in peripheral blood oxygen saturation from baseline.”
