# Supplementary material for: Health-Related Quality of Life and Sleep Quality after 12 Months of Treatment in Nonsevere Obstructive Sleep Apnea: A Randomized Clinical Trial with Continuous Positive Airway Pressure and Mandibular Advancement Splints
Source: Int J Otolaryngol. 2020 Jun 30;2020:2856460. doi: 10.1155/2020/2856460 (PMC7349617; doi:10.1155/2020/2856460)
Supplement: Supplementary Materials — Supplementary tables presenting SF36 domain scores at baseline and follow-up according to the 0−100 scales. Intention-to-treat analyses (Table S1), per-protocol analyses (Table S2), and complete case analyses (Table S3) are provided. Table S4: present complete case analyses of the norm-based SF36 domain scores and PSQI global score at baseline and follow-up. [file 2856460.f1.pdf]

Supplementary Materials

Supplementary tables presenting SF36 domain scores at baseline and follow-up according to the 0-100 scales. Intention-to-treat analyses (Table S1), per protocol analyses (Table S2) and complete case analyses (Table S3) are provided. Table S4 present complete case analyses of the norm-based SF36 domain scores and PSQI global score at baseline and follow-up.

Supplementary Table S1 SF36 domains (0-100 scales) at baseline and final follow-up (12 months), intention-to-treat.

	**Baseline**		**Final follow-up**		
**SF36 domains**	CPAP (n=55)	MAS (n=49)	CPAP (n=55)	MAS (n=49)	P
**Physical functioning**	83.7 (16.7)	81.6 (16.1)	87.1 (15.6)	83.0 (18.5)	.22
**Role-physical**	75.7 (25.0)	70.9 (31.1)	82.2* (22.7)	75.0 (30.8)	.19
**Bodily pain**	68.4 (29.1)	60.2 (26.8)	70.5 (27.3)	61.7 (29.6)	.12
**General health**	65.4 (20.6)	63.9 (22.8)	69.6 (20.9)	66.1 (23.5)	.43
**Vitality**	46.7 (20.7)	40.1 (19.5)	55.5* (20.8)	55.4* (21.0)	.98
**Social functioning**	77.0 (24.8)	71.9 (24.5)	83.2 (24.3)	80.6 (20.6)	.57
**Role-emotional**	84.7 (21.7)	84.4 (21.9)	87.6 (23.1)	87.6 (19.8)	1.00
**Mental health**	78.2 (13.9)	78.1 (15.8)	79.7 (16.3)	81.6 (12.0)	.50

CPAP=Continuous Positive Airway Pressure
MAS=Mandibular Advancement Splint
SF36 domains: Mean (standard deviation)
P: t-test between MAS and CPAP treatment groups at final follow-up
*Statistically significant change from baseline to final follow-up within treatment group, paired t-test ($P<0.05$)

Supplementary Table S2 SF36 domains (0-100 scales) at baseline and final follow-up (12 months), per protocol (compliant patients only).

	**Baseline**		**Final follow-up**		
**SF36 domains**	CPAP (n=18)	MAS (n=36)	CPAP (n=18)	MAS (n=36)	P
**Physical functioning**	83.1 (16.9)	84.0 (14.1)	86.4 (15.5)	86.5 (16.1)	.98
**Role-physical**	74.3 (22.9)	71.4 (33.0)	78.1 (26.1)	78.0 (30.0)	.98
**Bodily pain**	66.2 (31.4)	62.0 (26.8)	64.2 (30.0)	62.8 (29.2)	.87
**General health**	64.3 (21.6)	66.4 (22.3)	71.3 (22.4)	71.4 (21.7)	.98
**Vitality**	47.2 (25.5)	41.0 (19.2)	62.2* (18.0)	60.9* (16.3)	.80
**Social functioning**	81.3 (24.3)	71.9 (25.8)	91.7* (16.6)	83.7* (18.1)	.12
**Role-emotional**	87.5 (19.6)	83.6 (23.7)	92.6 (16.6)	89.1 (19.4)	.52
**Mental health**	80.3 (11.8)	77.5 (17.3)	85.8 (11.4)	83.5* (11.5)	.48

CPAP=Continuous Positive Airway Pressure
MAS=Mandibular Advancement Splint
SF36 domains: Mean (standard deviation)
P: t-test between MAS and CPAP treatment groups at final follow-up
*Statistically significant change from baseline to final follow-up within treatment group, paired t-test (P<0.05)

Supplementary table S3 SF36 domains (0-100 scales) at baseline and final follow-up (12 months), complete cases.

	**Baseline**		**Final follow-up**		
**SF36 domains**	CPAP (n=34)	MAS (n=39)	CPAP (n=34)	MAS (n=39)	P
**Physical functioning**	84.1 (15.5)	81.2 (17.0)	87.1 (13.9)	83.3 (19.8)	.35
**Role-physical**	74.1 (25.1)	69.6 (32.2)	80.7 (22.9)	75.2 (31.9)	.40
**Bodily pain**	65.1 (29.4)	60.2 (27.0)	68.9 (28.4)	60.6 (29.5)	.23
**General health**	63.9 (20.8)	64.3 (22.7)	69.2 (20.7)	69.5 (22.5)	.96
**Vitality**	46.0 (21.9)	39.1 (19.9)	58.1* (19.3)	58.7* (19.1)	.90
**Social functioning**	73.9 (25.1)	70.8 (25.5)	86.4* (22.1)	83.7* (19.9)	.58
**Role-emotional**	83.3 (22.2)	82.5 (22.8)	88.5 (19.4)	88.7 (18.8)	.97
**Mental health**	77.4 (13.9)	77.9 (16.7)	81.0 (15.0)	83.1* (11.7)	.52

CPAP=Continuous Positive Airway Pressure
MAS=Mandibular Advancement Splint
SF36 domains: Mean (standard deviation)
P: t-test between MAS and CPAP treatment groups at final follow-up
*Statistically significant change from baseline to final follow-up within treatment group, paired t-test ($P<0.05$)

Supplementary Table S4 SF36 domains (norm-based scales) and PSQI global score at baseline and final follow-up (12 months), complete cases only.

	Baseline		Follow-up			
**SF36 domains**	CPAP (n=34)	MAS (n=39)	CPAP (n=34)	MAS (n=39)	Adj. difference (95% CI)§	P§
**Physical functioning**	48.4 (8.3)	47.1 (8.7)	49.9 (7.7)	48.2 (10.4)	-1.2 (-4.3 – 1.9)	.45
**Role-physical**	49.2 (6.4)	48.1 (8.6)	50.9 (6.4)	49.6 (8.7)	-1.6 (-4.9 – 1.7)	.34
**Bodily pain**	47.8 (11.7)	46.1 (10.3)	49.3 (11.2)	46.2 (11.4)	-1.8 (-6.3 – 2.8)	.44
**General health**	45.6 (9.9)	46.0 (10.6)	48.0 (9.8)	48.4 (10.4)	-0.1 (-3.8 – 3.6)	.96
**Vitality**	42.3 (11.8)	39.0 (10.2)	48.6* (10.3)	49.2* (9.5)	1.7 (-3.0 – 6.4)	.48
**Social functioning**	42.7 (12.9)	41.5 (12.7)	49.0* (11.5)	47.8* (10.0)	-0.6 (-5.6 – 4.4)	.81
**Role-emotional**	47.8 (8.4)	47.7 (8.4)	49.7 (7.4)	49.9 (7.1)	0.6 (-2.6 – 3.8)	.72
**Mental health**	47.0 (10.3)	47.6 (12.0)	49.7 (10.9)	51.3* (8.3)	1.1 (-3.0 – 5.3)	.59
**Physical Component Score**	48.4 (7.4)	46.8 (9.6)	49.8 (7.5)	47.3 (10.7)	-1.3 (-4.2 – 1.6)	.38
**Mental Component Score**	44.6 (11.6)	44.3 (12.2)	49.2* (10.4)	50.6* (8.1)	2.1 (-1.9 – 6.1)	.29
**PSQI global score**	7.7 (3.4)	8.0 (3.3)	6.4* (2.7)	5.8* (2.6)	-0.8 (-1.8 – 0.1)	.09

CPAP=Continuous Positive Airway Pressure
MAS=Mandibular Advancement Splint
PSQI=Pittsburgh Sleep Quality Index
SF36=Medical Outcomes Study Short-Form 36-Element Health Survey
SF36 domains and PSQI global score: Mean (standard deviation)
*Statistically significant change from baseline to follow-up within treatment group, paired t-test ($P<0.05$)
§Difference between MAS and CPAP treatment groups at follow-up, based on linear regression analysis adjusted for baseline variables (Age, BMI, sex, smoking, baseline AHI, and the baseline SF36 domain/PSQI global score), reference group: CPAP
